# Screening, assessment and management of perioperative malnutrition: a survey of UK practice

**DOI:** 10.1186/s13741-021-00196-2

**Published:** 2021-08-26

**Authors:** L. S. Matthews, S. A. Wootton, S. J. Davies, D. Z. H. Levett

**Affiliations:** 1 Department of Perioperative Medicine, University Hospital Southampton NHS Foundation Trust, Southampton, SO16 6YD UK; 2 University of Southampton, Southampton, UK; 3 National Institute for Health Research Cancer and Nutrition Collaboration, Southampton, UK; 4 Department of Dietetics, University Hospital Southampton NHS Foundation Trust, Southampton, UK

**Keywords:** Nutritional risk, Preoperative evaluation, Post-operative complications, Perioperative pathways, Prehabilitation

## Abstract

**Background:**

Perioperative malnutrition is common and is associated with increased mortality, complications and healthcare costs. Patients having surgery for cancer and gastro-intestinal disease are at particular risk. It is a modifiable pre-operative risk factor and perioperative clinicians are well placed to identify those at risk and instigate interventions shown to improve outcome. Thus, we conducted a survey of Perioperative Medicine Leads with the aim of assessing the current provision of nutritional screening and intervention pathways in the UK.

**Methods:**

Perioperative Medicine Leads registered with the Royal College of Anaesthetists were asked to complete an online survey exploring current practice in screening, assessment and management of malnutrition in the perioperative period. The survey included a mixture of open and closed questions, graded response questions and options for free text. Where a response was not received, departments were phoned directly and e-mails sent to non-responders.

**Results:**

We received 121 completed questionnaires from 167 Perioperative Medicine Leads (response rate of 72.5%). Seventy respondents (57.9%) reported using the Malnutrition Universal Screening Tool to screen patients; however, only 61 (50.4%) referred patients at nutritional risk onto a dietitian. Sixty (49.6%) lacked confidence in local ability to identify and manage malnutrition perioperatively, with 28 (23.1%) reporting having a structured pathway for managing malnourished patients. One hundred eleven respondents (91.7%) agreed that malnutrition impacts on quality of life after surgery and 105 (86.8%) felt adopting a standard protocol would improve outcomes for patients. Those reporting a lack of confidence in dealing with malnutrition perioperatively cited a lack of organisational support, patients being seen too close to surgery and lack of clarity around responsibility as key reasons for difficulties in managing this group of patients.

**Conclusions:**

Malnutrition in the perioperative period is a modifiable risk factor which is common and results in increased morbidity for patients and increased cost to healthcare systems. This survey highlights areas of practice where perioperative clinicians can improve the assessment and management of patients at nutritional risk prior to elective surgery.

**Supplementary Information:**

The online version contains supplementary material available at 10.1186/s13741-021-00196-2.

## Background

Malnutrition in the perioperative period is associated with increased morbidity, mortality, length-of-stay and healthcare costs (Weimann et al. 2017). Pre-operative malnutrition is common and is estimated to have a prevalence of up to 65% in patients undergoing surgery for cancer or gastro-intestinal disease (Wischmeyer et al. 2018). These patients are at particular risk due to inadequate oral intake, cancer cachexia, muscle protein depletion and systemic inflammation (West et al. 2017; Arends et al. 2017; Bozzetti et al. 2007; Correia et al. 2001)_._

Optimising nutrition pre-operatively has been shown to improve outcomes after surgery (Pan et al. 2013; Vaid et al. 2012) and may impact long-term health outcomes (Horowitz et al. 2015). The extent to which perioperative clinicians include screening for malnutrition and pathways for the nutritional assessment and management of patients identified as at risk is unclear. We conducted an online survey amongst Perioperative Medicine Leads to determine the current provision of nutritional screening and intervention pathways in the UK.

## Methods

We did not seek Research and Ethics Committee approval for this survey as it was voluntary, an overview of current practice, and did not involve patient contact or information. After the survey had closed, respondents were contacted by the Centre for Perioperative Care to ask if there were objections to publication of the anonymised results. No objections were received.

Perioperative Medicine Leads registered on the Royal College of Anaesthetists (RCoA) database in December 2018 were contacted by the Perioperative Medicine department at the RCoA by e-mail. They were asked to complete an online survey (Google Forms, Google, Mountain View, California, USA). The survey had been reviewed internally by the RCoA College Council prior to dissemination. If a response was not received, departments were phoned directly and e-mails sent to non-responders on up to three occasions. Children’s hospitals were excluded from the final analysis, leaving a denominator of 167 hospital trusts distributed across the UK.

Respondents were explicitly asked to discuss the survey with dietetic, nursing and surgical colleagues if necessary. We identified five areas of practice that we wanted to assess as part of the survey: (i) nutritional screening, (ii) assessment of malnutrition, (iii) management of patients identified as malnourished or at risk of undernourishment, (iv) attitudes around malnutrition in the perioperative period and (v) local barriers to implementing care.

The survey included a mixture of open and closed questions, graded response questions and options for free text. Respondents were also asked to use free-text comments to identify areas of good local practice (Supplementary information).

The survey data were exported from Google Forms (Google, Mountain View, California, USA) to Microsoft Excel (Microsoft Corporation, Redmond, Washington, USA) for further analysis.

## Results

We received 121 completed questionnaires from 167 Perioperative Medicine Leads in hospital trusts across the UK (response rate of 72.5%) between December 2018 and July 2019.

### Nutritional screening

Over 75% of respondents (94, 77.7%) indicated that pre-operative malnutrition screening was performed by nursing staff in their hospital. Other groups of healthcare professionals reported as performing screening were surgeons (26, 21.5%), anaesthetists (25, 20.7%) and dietitians (23, 19.0%). Fifteen respondents (12.4%) did not know who was responsible for screening, four (3.3%) stated that no one was responsible for screening and one respondent reported that a junior doctor was dedicated to collecting this information in their hospital.

The most widely reported approaches to screening for risk of malnutrition were BMI (75, 62.0%), MUST (70, 57.9%) and percentage weight loss (24, 19.8%). A variety of other tools were used less frequently or the respondent did not know which tools were used (Table 1).

**Table 1:** Screening tools used for identifying nutritional risk

Tool used to identify nutritional risk	Number of respondents
Body mass index	75 (62.0%)
Malnutrition Universal Screening Tool (MUST)	70 (57.9%)
Percentage weight loss	24 (19.8%)
Disease specific tool	2 (1.7%)
Malnutrition Screening Tool (MST)	2 (1.7%)
Short Nutritional Assessment Questionnaire (SNAQ)	2 (1.7%)
Nutrition Risk Screen 2002 (NRS-2002)	1 (0.8%)
None	3 (2.5%)
Did not know	14 (11.6%)

### Assessment of malnutrition in patients identified at risk by screening

The commonest reported anthropometric measure was BMI (104, 86.0%). Alternative anthropometric assessments included arm muscle circumference (4, 3.3%), skin-fold thickness (2, 1.7%) and hand-grip strength (1, 0.8%). Ten respondents (8.3%) stated that no anthropometric testing was undertaken and 10 (8.3%) did not know what local practice was.

Over a third of respondents (42, 34.7%) reported that no specific biochemical assessments were performed and 11 (9.1%) did not know what local practice was. One respondent stated that the use of biochemical assessment was “surgery dependent” and one used a “specific malnutrition screen”. As a marker of disease severity and inflammation, serum albumin was the most common biochemical assessment, reported by 74 respondents (61.2%). Other biochemical tests included total protein (25, 20.7%), CRP (15, 12.4%), transferrin (11, 9.1%) and lipid studies (8, 6.6%).

An assessment of body composition, such as bioelectrical impedance analysis or CT analysis, was only performed in two hospitals (1.7%). Ninety respondents (74.4%) stated that no body composition assessment was undertaken and 29 (24.0%) did not know what local practice was. One respondent stated that this was “patient dependent”.

Functional testing was undertaken by 57 respondents (47.1%), most frequently using a self-reported questionnaire (29, 24.0%), timed get-up-and-go (16, 13.2%) or stair climb (14, 11.6%). Four trusts (3.3%) assessed functional status using cardio-pulmonary exercise testing. Forty-nine respondents (40.5%) stated that no functional testing was undertaken and 15 (12.4%) did not know what local practice was.

### Management of malnourished patients

**Fig. 1:**
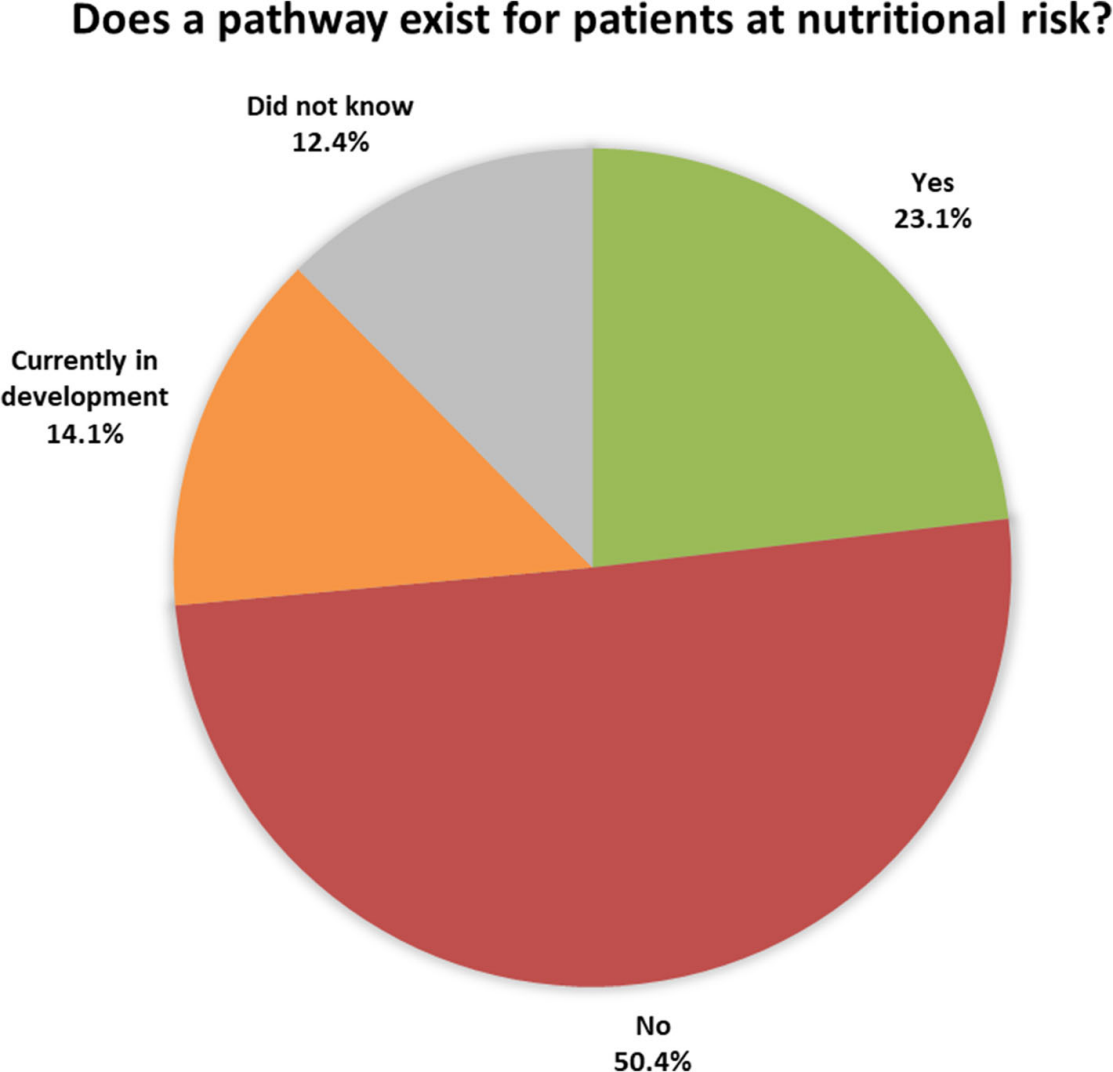
Local pathways for managing pre-operative patients at nutritional risk

Half of the respondents (50.4%) reported that their hospital had no perioperative pathway for managing malnourished patients. Twenty-eight respondents (23.1%) reported that their hospital did include a perioperative pathway for malnourished patients and 17 (14.1%) reported that their hospital was in the process of developing a pathway; 15 (12.4%) did not know whether or not a formal pathway existed (Fig. 1).

Where a patient was identified as being malnourished pre-operatively, 61 respondents (50.4%) reported that the patient would be referred to a dietitian. The remainder were referred on to other specialties, no one at all, or the respondent did not know whether an onward referral took place. (Table 2)

**Table 2:** Onward referral of malnourished patients

Specialty of onward referral	Number of respondents
Dietetics	61 (50.4%)
No one	12 (9.9%)
Surgical team	12 (9.9%)
General Practitioner	11 (9.1%)
Anaesthetic team	8 (6.6%)
Gastroenterology	2 (1.7%)
POM nutrition team	2 (1.7%)
Other	5 (4.1%)
Did not know	8 (6.6%)

Nearly half of respondents 47 (38.8%) reported that oral nutritional supplements were prescribed to malnourished patients in their hospital; 46 (38.0%) did not and 28 (23.1%) respondents were unsure of local practice.

### Attitudes around perioperative malnutrition (Fig. 2)

Nearly all of the respondents (111, 91.7%) either “agreed” or “strongly agreed” that malnutrition had an impact on quality of life following surgery; eight (6.6%) “strongly disagreed” and two (1.7%) neither “agreed” nor “disagreed”.

**Fig. 2:**
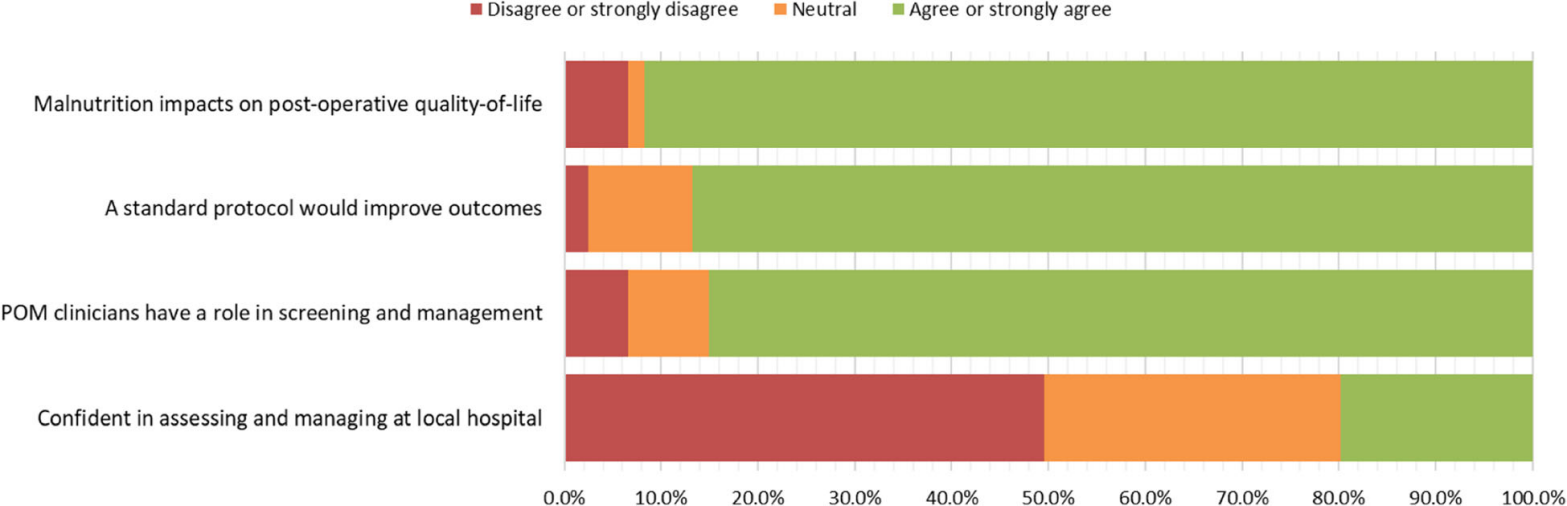
Attitudes around perioperative malnutrition

The majority of respondents (105, 86.8%) either “agreed” or “strongly agreed” that adopting a standard protocol for managing perioperative malnutrition would improve patient outcomes. Three “strongly disagreed” (2.5%) and 13 (10.7%) neither “agreed” nor “disagreed”.

The majority of respondents (103, 85.1%) either “agreed” or “strongly agreed” that perioperative clinicians had a role in the identification and management of malnutrition in the pre-operative period; eight (6.6%) either “disagreed” or “strongly disagreed” and 10 (8.3%) neither “agreed” nor “disagreed”.

Approximately, half of the respondents (60, 49.6%) either “disagreed” or “strongly disagreed” with the statement that “they were confident their hospital was able to identify and manage patients with malnutrition perioperatively”; 37 (30.6%) neither “agreed” nor “disagreed” and 24 (19.8%) either “agreed” or “strongly agreed”.

### Barriers to care

Those respondents who either “disagreed” or “strongly disagreed” with the statement that “they were confident their hospital was able to identify and manage patients with malnutrition perioperatively” (n = 60) were asked to detail their reasons for their disagreement. The main reasons given were: (i) lack of organisational support (58, 96.7%), (ii) patients seen too close to surgery (55, 91.7%), (iii) lack of clarity around responsibility (55, 91.7%), (iv) lack of training and education (38, 63.3%) and (v) time constraints (32, 53.3%).

## Discussion

This is the first survey of Perioperative Medicine Leads in the UK to evaluate the structured screening, assessment and management of malnutrition in patients undergoing elective surgery. The response rate of 72.5% suggests the survey is sufficiently representative of current practice.

Perioperative medicine is defined as the patient-centred, multidisciplinary and integrated medical care from the contemplation of surgery until full recovery. Consequently, an important part of the Perioperative Medicine Lead role should be to evaluate the pathways supporting nutritional screening and optimisation. Malnutrition is a modifiable risk factor and all patients’ nutritional status should be optimised before elective surgery (Weimann et al. 2017; Lobo et al. 2020).

We found that whilst the majority of Perioperative Medicine Leads indicated that patients were routinely screened for malnutrition, nearly half lacked confidence that their trust had an effective process for ensuring that all surgical patients were screened and treated in the perioperative period. The majority of hospitals are reported to be using the Malnutrition Universal Screening Tool for screening patients; however, only half are reported to refer patients identified as being at nutritional risk onto a dietitian and less than a quarter have a structured pathway for managing malnourished patients pre-operatively.

The Perioperative Medicine Leads in this survey overwhelmingly agreed that malnutrition impacts on a patient’s quality of life after surgery, that structured pathways for managing malnourished patients would improve outcomes, and that perioperative clinicians have a role in its management. However, those that lacked confidence in their hospital’s ability to identify and manage malnutrition in the surgical patient cited a lack of organisational support, proximity of seeing patients to surgery, lack of clarity around responsibility and inadequate training and education as reasons they felt they could not manage this group. So how do we bridge this gap between what we think we should be doing and reality?

### Screening for malnutrition

All patients having surgery should be screened for nutritional risk (National Institute for Health and Care Excellence 2017). It is the key first step in identifying those that may need additional support and is essential to avoid missing those who are malnourished without displaying overt symptoms. It is therefore surprising that only two-thirds of respondents stated the use of a screening tool validated for surgical patients. When a validated tool was used, this was almost exclusively MUST. Whilst MUST is recommended by the National Institute for Health and Care Excellence (NICE) for screening hospital and community patients, numerous other screening tools are available. For example, the Nutrition Risk Screening 2002 (NRS-2002) is recommended by European Society for Clinical Nutrition and Metabolism (ESPEN) (Weimann et al. 2017; Kondrup et al. 2003), yet only one respondent reported its use. Other examples include the Malnutrition Screening Tool, Short Nutritional Assessment Questionnaire and the recently developed Pre-operative Nutrition Score, which is a modification of MUST that also incorporates albumin (West et al. 2017). Regardless of which tool is being used, it is clear that the nutritional needs of some patients are not being appropriately addressed pre-operatively. We recommend that pre-operative pathways are mapped as a priority to identify the point of contemplation of surgery and that nutritional screening is performed as early as possible (Grocott et al. 2017). The screening tool used should be able to detect the presence of under-nutrition in an elective surgical population and should be standardised across all specialities to enable institution-wide consistency of practice.

### Assessment of the patient at risk of malnutrition

Any patient identified at being at risk of malnutrition should undergo a diagnostic assessment involving the identification of phenotypic (non-volitional weight loss, low BMI, low muscle mass) and aetiological (reduced intake, disease burden/inflammation) criteria (Cederholm et al. 2019). Whilst some of these criteria are included in many screening tools, it is crucial to highlight that screening and assessment are temporally different processes and confusing the two may result in misdiagnosis and inappropriate treatment. With specific regard to muscle mass, virtually no anthropometric or body composition assessments appear to be being performed in patients identified at nutritional risk. This finding is important as patients with higher lean body mass cope better with surgery, have fewer complications and spend less time in hospital (Kyle et al. 2005; Pichard et al. 2004; Van Venrooij et al. 2012).

Disease burden and inflammation are harder to objectively define. In the absence of a better test hypo-albuminaemia (albumin < 30g/l without hepatic or renal dysfunction) may be the best biochemical marker currently available and was used by nearly two-thirds of those surveyed. It is important that the perioperative clinician is aware that albumin reflects disease severity and related catabolism, and is not a direct measure of malnutrition. However, it is prognostic for complications and recommended by ESPEN for use in surgical patients (Weimann et al. 2017).

Assessment of patients should ideally be undertaken by those with accredited professional training in nutrition, such as a registered dietitian or physician with specific responsibility for clinical nutrition. However, current dietetic resources are largely directed to supporting patients after surgery. Outside of specialities where there is a high risk of malnutrition, such as upper gastro-intestinal cancer, pathways for pre-operative optimisation may be under-resourced and lack specialist dietitian input. It was, therefore, unsurprising that only half of those identified to be at risk received onward referral to a dietitian, with the remainder seeing either a variety of other professions or no one at all. This variability will be multi-factorial and may reflect an under-resourced, unstructured, pathway. In keeping with this, less than half of Perioperative Medicine Leads reported having a pathway for managing malnourished patients despite the vast majority agreeing this would improve outcomes. One solution to better direct the use of available resources may lie in formalising pathways and re-engineering the patient’s perioperative journey such that those at risk are identified at the time of referral for surgery, assessed earlier, with sufficient time then afforded to the interventions required to improve modifiable risk factors such as malnutrition (Grocott et al. 2017).

### Management of malnourished patients pre-operatively

The aims of treatment of a malnourished surgical patient are to improve nutritional status, limit wasting and ultimately maximise resilience and functional recovery. Where this cannot be achieved by dietary advice and food alone, nutritional support may be required (oral nutritional supplements (ONS), enteral tube feeding and/or parenteral nutrition). We did not examine enteral tube feeding or parenteral nutrition but did explore ONS as this is a simple intervention that can be actioned pre-operatively. NICE recommends considering ONS in those who are malnourished or at risk of malnutrition (National Institute for Health and Care Excellence 2017) as ONS has consistently been shown to increase intake (Sobotka 2010), reduce post-operative complications (Waitzberg et al. 2006) and be cost-effective (Elia et al. 2016). ESPEN goes further, suggesting it to be obligatory for all malnourished cancer and high-risk surgical patients and patients not receiving adequate intake through normal food (Lobo et al. 2020). Given this, we were surprised that less than half of Perioperative Medicine Leads report that their hospital prescribes ONS for malnourished patients. When considering implementing ONS into a nutritional care plan, it should be noted that patients need to be educated about its benefits and consideration given to the provision of energy-dense (> 2 kcal/ml) formulations, as both improve compliance (Grass et al. 2015).

So how can we improve the care we provide for this group of patients? Screening, assessing and managing these patients is important because malnutrition is one of the few modifiable pre-operative risk factors that, if addressed early and treated appropriately, can affect post-operative outcomes (Stratton and Elia 2007; Jie et al. 2012; Garth et al. 2010). Malnutrition is under-recognised and under-treated (British Association of Parenteral and Enteral Nutrition 2009; Marcos et al. 2003). It causes increased postoperative morbidity, excess mortality and increased costs (Weimann et al. 2017; Schneider et al. 2004; Sorensen et al. 2008). Cancer patients are at particular risk due to the effects of malignancy on nutrient metabolism and delays in surgery due to the side-effects of neo-adjuvant treatments (Andreyev et al. 1998). As a case in point, a recent study of patients undergoing colorectal cancer surgery found that malnourished patients were more likely to be readmitted within 30 days (Gillis et al. 2015).

There are numerous issues highlighted by this survey, some easier to remedy than others. It is noteworthy that many are common to previous work undertaken in Europe and North America, adding to the evidence that this is a key area for improvement (Grass et al. 2011; Williams and Wicschmeyer, 2017). Screening is a simple intervention and we encourage all Perioperative Medicine Leads to introduce a standardised tool early in the surgical pathway as a priority, as the first stage of identifying the size of this unmet need. Ideally, all patients identified as at risk should be assessed by a dietitian, although there may be significant resource implications if hospital dietitians are primarily focused on in-hospital patients. Early identification is essential to ensure sufficient time for optimisation without delaying surgery. Involving dietitians in the development of standardised pre-operative pathways will facilitate the multidisciplinary teamwork and data collection required to ensure an adequately resourced service. An example of a potential solution may be the development and implementation of perioperative nutrition clinics (Williams et al. 2020), though further work is needed to assess feasibility in the UK healthcare setting.

Looking to the future, technology and advances in therapies should be embraced to improve the way we assess and manage perioperative malnutrition. Examples include using bioelectrical impedance analysis, cross-sectional imaging or ultrasound to assess muscle mass (Williams et al. 2019), for nutrition risk scores to be transferred seamlessly between primary and secondary care and for patients to take responsibility for their own nutritional health through the use of smart device applications. Nutrition therapy is also a key component of multi-modal prehabilitation, recently advocated for all people with cancer, alongside psychological and exercise interventions (Macmillan Cancer Support 2019). Nutritional prehabilitation alone or combined with exercise reduces length of stay by 2 days in colorectal cancer surgery patients and may result in faster return to pre-operative fitness (Gillis et al. 2018). The results of larger randomised controlled trials are awaited.

One of the main strengths of this study is the high response rate, implying it is representative of current practice. We focused on the areas of nutritional evaluation that are clearly within the domain of Perioperative Medicine Leads, namely standardised screening, pathway management and barriers to care rather than focusing on areas of specific dietetic expertise. As such, our findings are most relevant to the perioperative clinician seeing patients prior to elective surgery in the pre-assessment clinic. However, our study has weaknesses. We relied on Perioperative Medicine Leads being able to discuss questions with other members of the multi-disciplinary team, which may not have occurred. Furthermore, whilst we believe that Perioperative Medicine Leads should be aware of local polices and nutritional services available in their hospital, this may not always be the case.

In conclusion, we report that there are deficiencies in the screening, assessment and optimisation of nutritional status prior to elective surgery in the UK. There is an urgent need to implement standardised pathways to ensure the optimisation of a risk factor that we know is amenable to intervention in a realistic pre-operative time frame and that has important impact on surgical outcomes.

## Supplementary Information

**Additional file 1:.** POM Leads Perioperative Nutrition Survey

## Data Availability

The survey is available in the supplementary information files. The data analysed are available from the corresponding author on reasonable request.

## Abbreviations

BMI: Body mass index
ESPEN: European society for clinical nutrition and metabolism
MUST: Malnutrition universal screening tool
NICE: National institute for health and care excellence
NRS-2002: Nutrition risk screening 2002
ONS: Oral nutritional supplements
RCoA: Royal college of anaesthetists
