# Supplementary material for: Screening, assessment and management of perioperative malnutrition: a survey of UK practice
Source: Perioper Med (Lond). 2021 Aug 26;10:30. doi: 10.1186/s13741-021-00196-2 (PMC8390283; doi:10.1186/s13741-021-00196-2)
Supplement: Supplementary file 1 — Additional file 1:. POM Leads Perioperative Nutrition Survey [file 13741_2021_196_MOESM1_ESM.pdf]

POM Leads Perioperative Nutrition Survey

Dear POM Lead,

We are asking all hospitals in the UK to complete this short survey regarding UK practice in screening for, assessing and managing malnutrition in the perioperative period.

This is part of a wider piece of work examining attitudes, knowledge and variability in UK perioperative nutrition practices with the aim of improving patients' nutritional state from the point of referral to surgery to eventual discharge home.

We would like to get as complete a picture as possible so please discuss it with dietetic, nursing and surgical colleagues if necessary. You will need to answer each question before being able to move forward.

If you have any queries please e-mail L.Matthews@soton.ac.uk.

Thank you for your time.

Dr Lewis Matthews

NIHR Academic Clinical Fellow

On behalf of The University of Southampton Critical Care/Anaesthesia & Perioperative Medicine Research Unit

* Required

About your hospital

1. Which school of anaesthesia are you in? *

Mark only one oval.

- Birmingham
- Bristol
- East of England
- East Midlands
- KSS
- London: Barts
- London: Central
- London: Imperial
- London: South East
- London: St George's
- Mersey
- North West
- Northern
- Northern Ireland
- Oxford
- Scotland: North of Scotland
- Scotland: South East Scotland
- Scotland: Tayside
- Scotland: West of Scotland
- South West
- Stoke on Trent
- Wales
- Warwickshire
- Wessex
- Yorkshire and the Humber - North and East
- Yorkshire and the Humber - South
- Yorkshire and the Humber - West
- Other

2. Which hospital are you the POM lead for? *

____

Pre-operative Nutrition Screening

This section refers to the screening for malnutrition in your hospital.

3. Who is responsible for screening patients for malnutrition prior to surgery in your hospital? (Please check all that apply) *

Check all that apply.

- [ ] Anaesthetists
- [ ] Dieticians
- [ ] Nurses
- [ ] Surgeons
- [ ] I do not know

Other: [ ] ______________________________

4. Which malnutrition screening tools are routinely used prior to surgery in your hospital? (Please check all that apply) *

Check all that apply.

- [ ] BMI
- [ ] Malnutrition Universal Screening Tool (MUST)
- [ ] Mid-upper arm circumference (MUA-C)
- [ ] Mini Nutritional Assessment – Short Form (MNA-SF)
- [ ] Malnutrition Screening Tool (MST)
- [ ] Nutritional Risk Screening 2002 (NRS-2002)
- [ ] Patient Generated Subjective Global Assessment (PG-SGA)
- [ ] Short Nutritional Assessment Questionnaire (SNAQ)
- [ ] Weight history (eg. % weight loss over a fixed period)
- [ ] None
- [ ] I do not know

Other: [ ] ______________________________

Pre-operative assessment of malnutrition

This section refers to the methods in which malnutrition is assessed if it is identified by the screening process.

5. Which of the following anthropometric tests are used to assess malnutrition before surgery in your hospital? (Please check all that apply) *

Check all that apply.

- [ ] Arm muscle circumference
- [ ] BMI
- [ ] Measure of skin-fold thickness (E.g. Triceps skin-fold thickness)
- [ ] None
- [ ] I do not know

Other: [ ] ______________________

6. Which of the following biochemical tests are used to assess malnutrition before surgery in your hospital? (Please check all that apply) *

Check all that apply.

- [ ] Albumin
- [ ] Pre-Albumin
- [ ] Total protein
- [ ] CRP
- [ ] Lipid studies
- [ ] Transferrin
- [ ] None
- [ ] I do not know

Other: [ ] ______________________

7. Which of the following body composition tools are used to assess malnutrition before surgery in your hospital? (Please check all that apply) *

Check all that apply.

- [ ] Bioelectrical impedance analysis
- [ ] CT
- [ ] DEXA
- [ ] None
- [ ] I do not know

Other: [ ] ______________________

8. Which of the following functional tests are used to assess malnutrition before surgery in your hospital? (Please check all that apply) *

Check all that apply.

- [ ] Gait speed
- [ ] Hand-grip strength
- [ ] Self-reported physical function questionnaire
- [ ] Stair climb
- [ ] Timed get-up-and-go
- [ ] None
- [ ] I do not know

Other: [ ] ____________________

Managing malnourished patients

This section refers to the management of malnourished patients during the peri-operative period.

9. If a patient is identified as being malnourished before surgery, are there formal pathways for managing pre-operative malnutrition? *

Mark only one oval.

- ( ) Yes
- ( ) No
- ( ) No, but one is being developed
- ( ) I do not know

10. If a patient is identified as being malnourished in pre-assessment who are they referred onto? *

Mark only one oval.

- Dietetic team
- Gastro-enterology team
- GP
- Surgical team
- Managed by anaesthetic team
- Dedicated peri-operative nutrition team
- No-one
- I do not know
- Other: ____________________

11. If a patient is identified as being malnourished in pre-assessment are they started on any oral nutritional supplements? *

Mark only one oval.

- Yes
- No
- I do not know

Attitudes and barriers

Please indicate the extent to which you agree or disagree with the following statements

12. Pre-operative malnutrition has an impact on a patient's quality of life following surgery *

Mark only one oval.

- Strongly disagree
- Disagree
- Neither agree nor disagree
- Agree
- Strongly Agree

13. Perioperative physicians have a role in identifying and managing pre-operative malnutrition *

Mark only one oval.

- Strongly disagree
- Disagree
- Neither agree nor disagree
- Agree
- Strongly agree

14. Adopting a standard protocol for managing peri-operative malnutrition would improve patient outcomes in your hospital *

Mark only one oval.

- Strongly disagree
- Disagree
- Neither agree nor disagree
- Agree
- Strongly agree

15. I am confident that my trust is able to identify and manage patients with malnutrition pre-operatively *

Mark only one oval.

- ( ) Strongly disagree
- ( ) Disagree
- ( ) Neither agree nor disagree
- ( ) Agree
- ( ) Strongly agree

16. If you have answered 'Disagree' or 'Strongly disagree' to the previous question please tell us what you think the local barriers are to identifying and managing peri-operative malnutrition? (Please check all that apply)

Check all that apply.

- [ ] Acceptability of the screening process
- [ ] Clinical judgment seen as better than using a screening tool
- [ ] Lack of organisational support
- [ ] Lack of training and education
- [ ] Lack of working relationship with dietetic team
- [ ] Not seen as the role of surgical pre-assessment to assess malnutrition
- [ ] Patients seen too close to surgery for meaningful intervention
- [ ] Time constraints limit the ability to assess malnutrition
- [ ] Unclear who is responsible for managing perioperative malnutrition

Other: [ ] ______________________

Examples of good practice

Please share your positive experiences

17. If you have any examples of good practice in assessing and managing peri-operative malnutrition that you would be happy to share please detail them below.

This content is neither created nor endorsed by Google.

Google Forms
